# Multiple mating and a low incidence of cuckoldry for nest-holding males in the two-spotted goby, *Gobiusculus flavescens*

**DOI:** 10.1186/1471-2148-9-6

**Published:** 2009-01-08

**Authors:** Kenyon B Mobley, Trond Amundsen, Elisabet Forsgren, Per A Svensson, Adam G Jones

**Affiliations:** 1 Department of Biology, Texas A&M University, 3258 TAMU, College Station, TX 77843, USA; 2 Department of Biology, Norwegian University of Science and Technology, NO-7491 Trondheim, Norway; 3 Norwegian Institute for Nature Research, NO-7485 Trondheim, Norway; 4 School of Biological Sciences, Monash University, Melbourne, VIC 3800, Australia

## Abstract

**Background:**

A major question in behavioural ecology concerns the relationship between genetic mating systems and the strength of sexual selection. In this study, we investigated the genetic mating system of the two-spotted goby (*Gobiusculus flavescens*), a useful fish model for the study of sexual selection whose genetic mating system remains uncharacterized. We developed four polymorphic microsatellite markers and used them to conduct parentage analyses on 21 nests collected during the breeding season to examine the rates of multiple mating by males and to test for evidence of alternative mating strategies.

**Results:**

Results of this study indicate that male *G. flavescens *mate with multiple females and enjoy confidence of paternity. We detected only one instance of sneaking, so cuckoldry contributed a very small percentage (~0.1%) of the total fertilizations in this population. Nests were nearly full and males that maintain larger nests have higher mating and reproductive success, irrespective of body size.

**Conclusion:**

Overall, our investigation shows that *G. flavescens *is similar to other, related gobies in that the nests of care-giving males often contain eggs from multiple females. However, *G. flavescens *differs from other gobies in displaying an extremely low rate of cuckoldry. The study of ecological factors responsible for this important difference between *G. flavescens *and related species should be a fertile area for future work.

## Background

Patterns of mate acquisition and reproduction are fundamentally important to the study of behavioural ecology and evolutionary biology. Behavioural observations and molecular analysis have revealed a diversity of reproductive modes and mating systems within natural populations of vertebrates [1,2]. Studies of this nature have provided insight into mechanisms for pre- and post-copulatory mating behaviours, alternative mating tactics, sexual selection, and the evolution of traits related to reproduction [1,2]. Despite these advances, it is often not known why some species display alternative mating tactics such as sneaking, female mimicry and egg piracy, while closely related species do not [3,4]. However, recent comparative analyses have shed some light on the evolution of alternative reproductive tactics. For example, alternative reproductive tactics likely arise within species with territory defence and/or mate monopolization, such that individuals that would normally be excluded from mating enjoy some reproductive success by using an alternative strategy [3].

Once an alternative reproductive tactic invades a population, either a mixed evolutionary stable strategy or a conditional strategy in which the alternative reproductive tactic is physiologically or environmentally determined maintains the polymorphism [5]. For a few species scattered throughout the animal kingdom such as certain isopods [6], fishes [7], lizards [8] and birds [9], alternative reproductive tactics have a substantial genetic component. However the occurrence of alternative reproductive tactics appears to be phenotypically plastic in most species [5]. Alternative mating tactics may switch from one state to another based on characteristics of the environment or individual. For example, nest site availability [10], an individual's age [11], or body size [12,13] may affect the probability of adopting an alternative mating tactic. Additionally, the observation that populations and closely related species sometimes differ with respect to alternative mating tactics suggests that these behaviours can vary on a macroevolutionary scale [14].

Fishes display a staggering array of reproductive behaviours, so they are ideally suited for behavioural studies [15]. Male alternative reproductive tactics are common among fishes and range from parasitic spawning behaviours such as sneaking, egg piracy and female mimicry to cooperative breeding between satellite and territorial males [reviewed in [3,4,16-19]]. It appears that male alternative reproductive tactics have evolved from mate monopolization and back again multiple times in several groups of fishes, suggesting that the invasion and loss of male alternative reproductive tactics is a frequent and rapid occurrence [3]. It also appears that the evolution of male alternative reproductive tactics in certain lineages is aided by the presence of hormonal biochemical pathways that facilitate their evolution [3,18]. Although male alternative reproductive tactics are common in species of fishes with male parental care [4], the presence of male parental care appears not to be significantly correlated with the evolution of alternative reproductive tactics [3]. Rather, strong sexual selection on males likely drives the evolution of male alternative reproductive tactics as evidenced by the significant correlation between male sexually selected traits and the presence of male alternative reproductive tactics [3].

The present paper details the genetic mating system of the two-spotted goby, *Gobiusculus flavescens *(Fabricius 1779). Male alternative reproductive tactics including sneaking and female mimicry occur frequently in gobies, making them good candidates for the study of alternative reproductive tactics [e.g. [20-22]]. Recent behavioural studies within the *Pomatoschistus *clade or "sand goby group" [sensu [23]] suggests a high degree of diversity in male alternative reproductive tactics, ranging from sneaking in common (*Pomatoschistus microps*) and sand gobies (*P. minutus*) to the absence of sneaking in the marbled goby (*P. marmoratus*) [12,24,25]. It is unclear to what extent sneaking plays a role in the mating behaviour of *G. flavescens*. The two-spotted goby has recently become established as a model for the study of factors affecting the intensity of sexual selection [26], but genetic mating patterns, including the prevalence of alternative mating strategies, have not yet been characterized. A detailed genetic characterization of the two-spotted goby mating system hence will contribute to progress in understanding sexual selection in this species and to comparative studies of two-spotted gobies and other related taxa.

In this study, we applied microsatellite-based parentage analysis to two-spotted gobies to address several questions of potential importance to the study of sexual selection in this species. First, are male two-spotted gobies similar to other gobies in caring for eggs from multiple females simultaneously within nests? Second, does the genetic mating system of two-spotted gobies reveal evidence of alternative male mating strategies? Third, is there evidence of sexual selection among males, as evidenced by correlations between body size and male mating success or reproductive success?

## Results

### Microsatellite analyses

The four microsatellites developed in this study were polymorphic with 13–18 alleles per locus (Table 1). Heterozygosities were high for all loci and ranged from 0.758 to 0.848 (Table 1). We found no evidence of genotypic disequilibrium (Fisher's exact test: P > 0.05) and all loci except 2SG21 were in Hardy-Weinberg equilibrium after Bonferroni adjustment [27]. Locus 2SG21 displayed a significant deficit of heterozygotes (Fisher's exact test: P < 0.001) suggesting the presence of a null allele. Exclusion probabilities for parentage analysis ranged from 0.592 to 0.811 for each locus and the exclusion probability for all loci combined was high at 0.991 for one parent known with certainty and the second parent unknown (Table 1).

**Table 1:** Microsatellite loci assayed from adult *Gobiusculus flavescens*.

Locus	Primer sequence (5'-3')	Repeat Motif	Temp (°C)	*N*	*A*	*H*_O_	*H*_E_	Excl. prob
2SG-08	F: TGATGGTTCTTCTTTCAATATGC R: GCTGCTGGACACCTGAATTT	(GATA)_13_	58	33	18	0.848	0.908	0.811
2SG-17	F: GCTGCTGGACACCTGAATTT R: CGATCGCCTTTCAGTTTGAC	(CTAT)_11_	56	33	15	0.788	0.763	0.592
2SG-21	F: TGTAGGTGCCTTCCCCATTA R: GGACTCCTGCATCTCTGCAT	(GATA)_10_	58	33	16	0.758	0.842	0.685
2SG-55	F: CATACATGCGTGCTCAAAAA R: TGTCGGTATTGAAACATCCAA	(NTAT)_14 _^a^	59	31	13	0.839	0.821	0.650

^a^Actual sequence = (CTAT)_4_GTAT(CTAT)_2_(GTATCTAT)_2_(CTAT)_3_

Name of locus, primer sequence of the original cloned microsatellite, PCR reannealing temperature (°C), number of adults assayed (*N*), number of alleles per locus (*A*), observed (*H*_O_) and expected heterozygosity (*H*_E_) and exclusion probabilities (Excl. prob; given one parent known with certainty and the second parent unknown) are listed for each locus.

The departure of locus 2SG21 from Hardy-Weinberg equilibrium was corroborated by the detection of a null allele in the nest holding males of C03, C14 and C21. The null allele occurred infrequently (0.03) in all adult fish genotyped. The null allele manifested itself clearly as sets of embryos homozygous for each maternal allele with an absence of embryos possessing the expected heterozygous genotype comprised of both parental alleles. Paternal null alleles did not compromise the interpretation of the parentage data as they were easy to detect within the progeny arrays. Null alleles present in maternal lines would be less obvious to detect. Maternal nulls, if present, could result in an overestimation of multiple maternal genotypes in a nest. However, such misdiagnoses would be based on a single locus and would invariably involve homozygous offspring genotypes. In the present analysis, all inferences of multiple maternal genotypes in a nest were corroborated by at least three loci, thereby minimizing overestimation of mothers based on null alleles. *De novo *mutations and genotyping errors were relatively infrequent; occurring in only one offspring in each of seven nests (7 out of 902 embryos or 0.008).

### Mating behaviour

Twenty-two nests and attendant males were collected during sampling. Twenty of the nests were in or on blue mussel (*Mytilus edulis*) shells. The other two nests collected consisted of eggs laid in a crevice made by three small rocks (C14) and one artificial nest (C21). Because only one out of 30 artificial nests was occupied, it appears that the addition of artificial nests prior to the study did not affect the natural mating dynamics.

Clutches of eggs in collected nests were at all stages of development, ranging from recently laid (absence of pigmented eyespots on larvae), eyed (development of strong pigmentation in eyes of larvae), to larvae hatching at the time of collection. The time it took for eggs to hatch after they were brought back to the lab ranged from 0–10 days, with an average of 5.2 ± 0.6 days from the date of collection. From the onset of hatching, nearly all larvae in a nest hatched within a 24 hour period.

**Figure 1:**
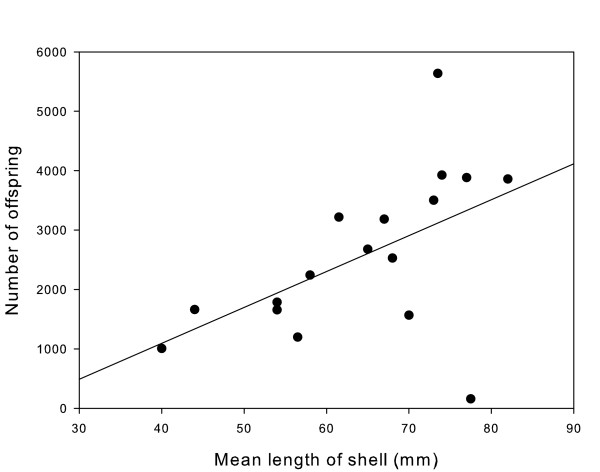
**Relationship between the number of offspring per nest and the mean length of shell for *Gobiusculus flavescens***. This relationship shows a positive and significant relationship between offspring and mean length of mussel shells (R^2 ^= 0.28, df = 16, P < 0.03).

Nests contained an estimated mean of 2296 ± 292 hatching larvae per nest (range 157 – 5636; Table 2). Actual clutch sizes were slightly larger due to the presence of undeveloped eggs and the potential of larvae to be lost during collection and rearing. The three nests that were in the process of hatching at time of collection (C1, C4 & C18), the artificial nest (C21), and the rock nest (C14) were excluded from the analyses involving nest (mussel) size. With two exceptions, nests in mussel shells were nearly full (89 ± 4%) with little extra room for additional eggs (Table 2). The number of offspring per nest was significantly correlated with the mean mussel shell length (ANOVA: F_1,15 _= 5.82, P < 0.03, Fig. 1). However, one nest where the male was spawning at the time of collection (C19) had particularly high leverage (Cook's distance > 0.5) due to the low number of eggs in the nest at the time. Removing this data point revealed an even stronger relationship between mussel length and number of offspring (ANOVA: F_1,14 _= 20.30, P < 0.0005). Male size, on the other hand, was not significantly related to mean mussel shell length (ANOVA: F_1,15 _= 0.37, P = 0.55).

**Table 2:** Summary of parentage analysis data for *Gobiusculus flavescens *nests

Male ID	TL (mm)	WM (g)	Nest ID	Nest fullness^a^	# larvae per nest	# larvae assayed	# females	# sneakers	# eggs sneaked
M01	48.0	0.79	C01	*---*	501	38	2	0	0
M02	40.5	0.48	C02	100	1005	42	3	0	0
M03	43.5	0.60	C03	90	3858	40	5	0	0
M04	44.5	0.64	C04	---	1789	81	4	0	0
M05	44.0	0.59	C05	80	2527	36	6	0	0
M06	41.5	0.50	C06	100	2241	41	4	0	0
M07	37.0	0.37	C07	100	3502	31	4	1	1
M08	46.0	0.74	C08^b^	90	1660	---	---	---	---
M09	42.5	0.48	C09	100	5636	42	5	0	0
M10	40.0	0.52	C10	100	3183	37	5	0	0
M11	45.5	0.60	C11	50	1565	44	3	0	0
M12	40.0	0.43	C12	100	3217	36	5	0	0
M13	41.5	0.57	C13	100	3925	70	6	0	0
M14	38.0	0.37	C14	---	852	26	4	0	0
M15	43.0	0.59	C15	90	1197	37	4	0	0
M16	40.0	0.40	C16	100	1655	30	4	0	0
M17	46.5	0.69	C17	100	3882	41	5	0	0
M18	45.0	0.67	C18	---	2946	38	6	0	0
M19	45.0	0.68	C19	30	157	32	2	0	0
M20	41.5	0.53	C20	100	1784	42	5	0	0
M21	43.0	0.64	C21	---	754	46	3	0	0
M22	38.0	0.47	C22	90	2676	72	5	0	0

^a^Nest fullness was not estimated in non-mussel nests (C14, C21) or in nests hatching at time of collection (C01, C04, C18).

^b^Parentage analysis could not be completed on nest C08 due to poor PCR amplification.

Total length (TL) and wet body mass (WM) are shown for each male surveyed and the nest ID, percent fullness of the nest (nest fullness), number of offspring per nest (# larvae per nest), number of offspring genotyped (# larvae assayed), number of unique reconstructed female genotypes (# females), the number of sneaker males detected (# sneakers) and the number of assayed larva that were fertilized by sneakers (# sneaked).

**Figure 2:**
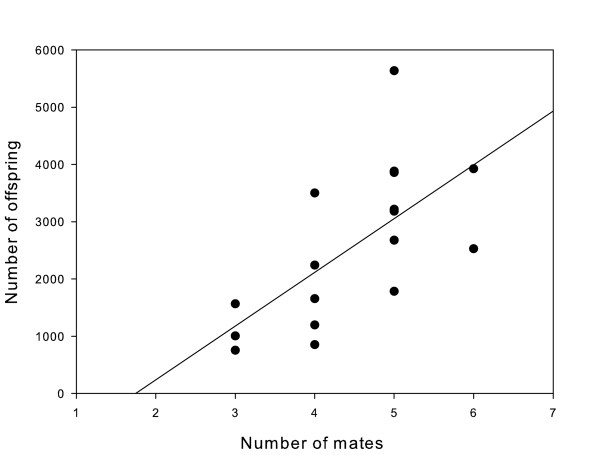
**Relationship between reproductive success and mating success of nest-holding male *Gobiusculus flavescens***. This relationship shows a positive and significant relationship between offspring and mates (R^2 ^= 0.41, df = 17, P < 0.004).

Parentage analysis was conducted on a sample of 26–81 (mean 40 ± 3) larvae per nest, in order to estimate the mating and reproductive success of males and to detect the presence of male alternative reproductive tactics (Table 2). We detected an average of 4.3 ± 0.3 mothers for each nest (n = 21) with a range from 2–6 females per nest (Table 2). The number of mothers was positively related to the number of offspring in a nest (ANOVA: F_1,16 _= 11.35, P < 0.004; Fig. 2). For this analysis, nests that were in the process of hatching (n = 3) were excluded as some offspring may have been lost. One nest (C13) appeared to have two attendant males but only one of the males' genotypes matched all offspring, so the matching male was assigned as the father for that particular nest. The same nest (C13) contained two broods of eggs that hatched nearly 48 hours apart. In this nest, we detected six maternal genotypes; three females with shared genotypes between the two broods, two unique female genotypes from the early brood, and one unique female genotype from the late brood (Table 2). Undeveloped eggs accounted for a small proportion of the total number of eggs within each nest. The number of undeveloped eggs ranged from 0–3.7% with a mean of 0.89 ± 0.24% per nest.

In the 21 nests surveyed, only one genotyped embryo within one nest satisfied our criteria for a sneaked copulation (C7, Table 2). In this instance, the genotype of the sire was not consistent with three of the four possible paternal alleles, but the larva's genotype was an exact four locus match to a particular reconstructed female genotype for that nest. The one larval allele that matched an allele of the collected father is common (0.29) and therefore the probability that the sneaker shared this allele with the resident male is relatively high. The sneaked copulation constituted 3.2% of the examined larvae in that nest, corresponding to an estimate of 113 larvae, in total. In all other nests, the genotypes of attendant males were consistent with all offspring surveyed. Thus successful sneaking appears to be a rare event during the sampled time period.

Attendant males had a mean total length (TL) of 42.5 ± 0.6 mm and had a mean wet body mass (WM) of 0.56 ± 0.03 g (Table 2). Male TL was significantly related with WM (ANOVA: F_1,17 _= 120.70, P < 0.0001). Neither of the two proxies of male fitness, TL nor WM, were correlated with either male reproductive or mating success at the sampled time period. Male mating success was not related to either male TL (ANOVA: F_1,19 _= 1.04, P = 0.32) or WM (ANOVA: F_1,19 _= 0.92, P = 0.35). Likewise, male reproductive success was not related to either male TL (ANOVA: F_1,16 _= 0.02, P = 0.49) or WM (ANOVA: F_1,16 _= 0.20, P = 0.66).

Females mothered between 48–2081 larvae per nest with an average of 549 ± 46 larvae. GERUD found 89 unique female genotypes reconstructed from progeny arrays and no reconstructed genotype was an exact four locus match for another reconstructed genotype. The probability of identity of females was low (1.3 × 10^-6^), indicating that the likelihood of sampling two unrelated females sharing an exact four locus genotype would be extremely small. The lack of matching female genotypes suggests that the local breeding population of *G. flavescens *at this site is quite large, such that the chance of encountering the same female mated to more than one male is low.

## Discussion

This study provides the first insights into the genetic mating system of the two-spotted goby. Based on microsatellite DNA analyses, we detected multiple female partners for all nest-holding males, with typically four females spawning with each male. The results of this study also demonstrate that *G. flavescens *males enjoy a high confidence of paternity with only one case of low frequency (~3%) sneaking discovered in the 21 nests surveyed. Although sneaking may be possible in *G. flavescens *below our threshold of detection, it would amount to a very small proportion of total offspring per nest. This very low frequency of sneaking is interesting given that several closely related species commonly display male sneaking behaviour [12,24].

In addition to sneaking, multiple paternity within two-spotted goby nests may also be caused by nest takeovers, a common phenomenon in closely related gobies such as sand gobies [28]. However, a nest takeover is an unlikely explanation for our data. First, the number of offspring sired by the non-resident male was very low. Second, the maternal genotype of the sneaked offspring matched other offspring of that nest, which were sired by the resident male. Although females are potentially capable of dividing their eggs between different nests (K de Jong, unpublished data), we never found the same reconstructed female genotypes in more than one nest. This suggests that finding the same female mating with a different male would be highly unlikely within this population.

What might account for the low rate of cuckoldry in two-spotted gobies relative to close relatives? One possibility is that two-spotted gobies exhibit temporal variation in rates of alternative mating strategies and that we happened to sample during a period of exceptionally low sneaking. This explanation may be especially germane to two-spotted gobies, as their sex roles have been documented to reverse during the breeding season [26]. Although the positive correlation between number of mates and reproductive success strongly suggest sexual selection occurs between males, two-spotted goby males are unlikely to compete strongly for mates during sex-role reversal [29]. Two lines of evidence from our data support the previous finding [29] that male mating competition is weak during the late part of the breeding season. First, the vast majority of nests maintained in mussel shells were nearly full suggesting that most males are not limited by access to mature females during this time. Second, there was no significant relationship between nest size and male body size, suggesting that males of any size may occupy nests. It then appears that male reproductive success is mainly limited by space within the nest, as evidenced by the strong correlation between shell size and the number of offspring produced. Thus, an interesting question, beyond the scope of the current study, is whether or not male reproductive behaviours we observed in the latter part of the mating season are similar to mating patterns in the early part of the breeding season.

### Male nest size

In this study, male size was unrelated to mussel shell size even though larger mussel shells were clearly advantageous with respect to mating and reproductive success of the resident males. Thus, all else being equal, we would expect males to prefer large mussel shells in order to maximize their fitness, similar to studies that have shown a clear relationship between male size and nest size in other gobies [30,31]. We can envision at least two scenarios that may explain the lack of a relationship between male size and mussel size. First, suitable mussel shells may be limiting, thereby forcing males to take up the first available nest they find, irrespective of size of nest. Second, males may simply not have a preference for large mussel shells, or any such preference may be trumped by more important nest characteristics. For example, gap of the opening between mussel shell halves may be important in *G. flavescens*. In a related species, *Pomatoschistus minutus *(sand goby), females prefer males with the smaller nest openings as it likely deters predation and potential sneaking [32,33]. This second hypothesis suggests that there is a trade off between nest size and other environmental variables related to fitness. Additional studies are needed to elucidate what factors contribute to male nest choice in male *G. flavescens*.

### Female clutch size

The maximum reproductive success of an individual female was 2081 larvae, which is similar to a maximum of 2101 eggs reported in a previous laboratory study [34]. However, the average reproductive success was much lower than in earlier studies. When wild-caught females are placed with a single male in a laboratory setting, they laid on average 1368 ± 40 [34] and 1287 ± 36 eggs [35], compared to 549 ± 36 larvae per female in this study. Thus, there is a nearly three fold reduction in the mean number of offspring per female in this study compared to earlier studies. Because this difference in mean female reproductive contribution may lead to different interpretations of the genetic mating system, we explore potential causes of this discrepancy here.

Eggs failing to develop are unlikely to explain this discrepancy, as our nests had a low proportion of undeveloped eggs (< 4%), on par with earlier investigations showing high hatching success (> 90%) when the male is excluded from tending the nest [35]. Clutches harvested in the field contained eggs from several females laid in an asynchronous manner. In this way, some eggs from older clutches may have hatched prior to collection, underestimating a female's reproductive contribution to a particular clutch of eggs. However, since most eggs in nests hatched within 24 hours of each other, the potential for partially hatched clutches does not appear to play a major role in the small clutch sizes of individual females. Paternal filial cannibalism, i.e. predation by males on a portion of the eggs in the nest, may also partly explain the low mean female reproductive success. In the laboratory, *G. flavescens *males typically consume slightly less than a third of the eggs in their nest [36]. However, the degree of filial cannibalism under presumably harsher natural conditions is currently unknown for this species. Other environmental influences, such as disease and egg predation, may also influence female reproductive success, but the frequency and severity of these events are unknown in the wild.

Another possible contributor to the clutch size discrepancy is physical interference between females during mating. Such physical female interference during copulation is known in fish [e.g. [37]] and may be common in polygynous mating systems [38]. In the field, there is the potential for physical competition between *G. flavescens *females, especially late in the breeding season when males are often courted by as many as 15 females simultaneously [26]. It is important to note that the laboratory studies reporting large clutch sizes by females mated single males with a single female [34,35]. Thus, there was no potential for interference from other females during spawning. Competition for egg-laying space within the nest may be another important factor in reducing the mean female reproductive contribution. As a nest becomes increasingly full, a female would face the choice of either contributing only few eggs in the remaining spaces, or clearing an area by cannibalizing the eggs of a previous female. Most nests collected were either completely filled with eggs, or nearly so (Table 2), indicating that competition for suitable egg-laying substrate within a male's nest is high.

### Male alternative reproductive tactics in the "sand goby" group

*Gobiusculus flavescens *belongs to the *Pomastoschistus *clade or "sand goby" group, a monophyletic group of gobies common in the eastern Atlantic Ocean and the Baltic and Mediterranean seas [23]. Molecular clock estimations suggest that this group diversified in a fairly short time period during or after the Messinian salinity crisis at the end of the Miocene (4–4.5 mya). Members of this clade likely evolved from a single common ancestor that occupied a benthic marine lifestyle.

For species investigated within the sand goby group, there appears to be a wide range of behaviours associated with sneaking and male alternative reproductive tactics. At least two species display male alternative reproductive tactics in the form of sneaked copulations, *P. microps *(common goby) [12,39,40] and *P. minutus *[24,41] and at least one species, the marbled goby *P. marmoratus*, appears to lack male alternative reproductive tactics as judged by gonadal analyses [25]. Sexual selection regimes vary in both the common goby and the sand goby as a result of differences in nest-site availability, the operational sex ratio, and temperature [41-45]. A study of male alternative reproductive tactics in *P. minutus *revealed that sneaker males parasitized approximately 50% of nests surveyed amounting to nearly 11% of all fertilizations on average [24]. A second study revealed surprisingly similar sneaking rates between populations of *P. minutus *that had a large difference in the availability of suitable nest sites [41]. Similarly, experimental manipulations of nest-site availability in *P. minutus *showed that male sneaking behaviour occurred frequently and was resilient to changes in nest-site availability [45]. Unlike the sand and common goby, the marbled goby, *P. marmoratus*, is speculated to lack male alternative reproductive behaviours based on estimated gonadosomatic and seminal vesicle somatic indices [25]. Both of these measures suggest that investment in gonads relative to body size is constant, suggesting that males lack sneaker morphology. Although conclusive parentage analysis have not yet been performed for the marbled goby or the common goby, these observations hint at an interesting scenario in which male alternative reproductive tactics are important and maintained in certain lineages (sand goby, common goby) but not in other lineages such as the two-spotted goby and the marbled goby. The resolution of this question awaits a systematic classification of the genetic mating system of all members of the *Pomatoschistus *clade and a comparison of the ecological and evolutionary factors that may influence male alternative reproductive tactics within individual species such as *G. flavescens*.

## Conclusion

Understanding the evolutionary consequences of alternative reproductive behaviours is a fundamental goal of evolutionary biology. In this study we characterized the genetic mating system of a species that serves as a model species for behavioural ecology. Our results demonstrate a high incidence of multiple mating and a remarkably low sneaking rate among nest-holding males in comparison with closely related species. Additional investigations of the genetic mating system in this and other lineages are clearly warranted to elucidate the relationship between the strength of sexual selection and the evolution of male alternative reproductive tactics.

## Methods

### Study species

*Gobiusculus flavescens *is a small, semi-pelagic marine fish that inhabits shallow waters along rocky shores of the northwestern Atlantic from Portugal to Norway. During the breeding season, males maintain nests in empty bivalve shells, rocky crevices and brown algae [46]. Courtship can be initiated by either the male or the female and courtship behaviours of males include fin displays, quivers and leads, whereas females court by performing a sigmoid display [26,34,47]. After a female lays her clutch, the male fertilizes the eggs and provides the sole parental care of developing embryos until hatching [48]. Within a single breeding bout, a female can lay a clutch of 1000–1500 eggs [34,35] and several females have been observed to lay their eggs in the nest of the same male [46]. Larval hatching is dependent on ambient water temperatures and hatching time can range from one to more than three weeks [36,48]. Both males and females likely reproduce several times during the breeding season [46]. Males have been observed in and around other males' nests but sneaked copulations have not been documented in the field and males do not appear to have any dimorphism in color or size suggesting a sneaker morph in this population (K.B. Mobley, E. Forsgren & T. Amundsen, personal observation).

### Field collections and nest hatching

All nests and attendant males were collected inside Gåsevik, a shallow (0.5 – 3.0 m) bay approximately 40 m × 60 m wide and situated near the Kristineberg Research Station at the mouth of the Gullmar fjord on the West Coast of Sweden (N58°14.778', E11°26.144'). The benthos inside of the bay is principally comprised of small rocks and blue mussels (*Mytilis edulis*) and supports a high density of natural nest sites (K. Mobley and S. Wacker, personal observation). Also scattered throughout the bay are larger rocks covered with live blue mussels and algae and small patches of seagrass. The edges of the bay are rocky outcrops supporting high densities of live blue mussels and algae. We placed 30 artificial nests consisting of an 80 mm long, 25 mm diameter PVC tube, lined with a clear acetate sheet, attached to a stone weight, on July 4. These nests were added to increase the chances of locating, observing and capturing males and these nests generally have high occupancy early in the season (T. Amundsen and E. Forsgren, personal observation).

Collections took place on nine occasions between July 4 and July 22, 2005, using either snorkel or SCUBA gear. Males that appeared to be stationary (i.e. nest holding) were observed for five to 15 minutes until they entered the nest, so that the position of the nest could be determined. After capturing the attending male using hand nets, the nest was collected. If the nest did not contain any eggs, the male was released. Nests and males were then transported live in plastic containers to Kristineberg Marine Research Station. At the station, the attendant males were measured for total length (TL, tip of snout to tip of tail) to the nearest 0.5 mm and wet body mass (WM) was ascertained to the nearest mg. Males were then sacrificed by severing the spinal column above the operculum and placed in 95% EtOH for genetic analysis. An additional 30 adult females and 10 adult males were collected from the same bay at the end of the study to estimate population allelic frequencies of microsatellite loci. This collection represented a small portion of the adult female population size as females can outnumber males nearly 10:1 in the late season [26].

Three of the clutches were hatching during the collection of nests, so the larvae from these nests were immediately preserved in 95% EtOH. Remaining nests were photographed and the length of each mussel shell was recorded to the nearest mm with calipers. The length of mussel shells were calculated as the maximum distance from the umbo (the narrow part of the shell near the hinge) to the outer edge of the shell, and was averaged for both shell halves. The percentage of the nest containing eggs (nest fullness) was estimated by eye from photographs in 10% increments. Eggs are laid inside shells and are generally absent near the umbo and along the outer edge of inside of the shell. Therefore these areas were not included in the visual estimation of nest fullness. Nests were placed in l5 l incubation aquaria supplied with aerated fresh seawater [35]. Aquaria were maintained at 16–18°C and inspected each day for larval development and hatching. Hatched larvae were collected over a 24 hr period by straining incubation tank water through a 90 μm sieve and fixed in 95% EtOH. Remaining undeveloped eggs (opaque in appearance) were removed from the nest substrate and combined with the hatched larvae. In one instance (C13), some eggs hatched while others were still in early development. In this case, we allowed the remaining eggs to develop for an additional 48 hrs before collection of the second batch of larvae.

### Microsatellite development and analysis

Microsatellite markers were isolated from a single *G. flavescens *adult using the microsatellite development protocols described by Ardren et al. [49] and modified by Hoffman et al. [50]. Briefly, DNA was isolated from *G. flavescens *using a standard proteinase K, phenol-chloroform procedure [51]. An enriched microsatellite library was constructed using a modification of a biotinylated oligonucleotide procedure originally described by Kijas et al. [52]. Degenerate oligonucleotide-primed polymerase chain reaction (DOP-PCR) conducted with the K6-MW primer [53] was used to generate small DNA fragments with known flanking sequences. Amplification of fragments was accomplished in 50 ul reactions and PCR cocktails were identical to those of Ardren et al. [49] with approximately 50–100 ng of *G. flavescens *genomic DNA. The following PCR temperature profile was used for the DOP-PCR: 95°C for 2 min; five cycles of 95°C for 30s, 30°C for 1.5 min, ramp at 0.2°C/s to 72°C and 72°C for 3 min; 29 cycles of 95°C for 30s, 56°C for 1.5 min, 72°C for 3 min; 72°C for 20 min.

Fragments generated using the DOP-PCR were enriched for a (GATA)_8 _repeat motif using a biotin/streptavidin (Promega) enrichment procedure. Hybridization conditions were identical to Ardren et al. [49] except the hybridization temperature was cooled from 98°C to 60°C by a rate of 1°C/s and held at 60°C for 25 min and the final washes were done at 71°C. A second DOP-PCR was conducted using the enriched DNA and 4 ul of the resulting PCR product were used to clone the product using a Topo TA cloning kit (Invitrogen). Of the 672 positive clones screened, 60 positive clones were identified and sequenced using the T3 primer at Nevada Genomics (Reno). Of the sequenced samples, 29 contained repeat motifs, nine of which were unique. Primer pairs were designed with Primer3 version 0.4.0 [54] using program defaults.

Microsatellite markers were amplified using polymerase chain reaction (PCR) in a 20 ul volume containing 1× PCR buffer, 1.75 mM MgCl_2_, 0.2 mM of each dNTP, 0.15 uM of each primer, 0.5 units of *Taq *polymerase, and 2 ul of genomic DNA. Temperature profiles for thermal cycling were as follows: 92°C for 1 min; 35 cycles of 92°C for 1 min, 1 min at the optimal annealing temperature (Table 1), 2 min at 72°C; and a final 4 min extension at 72°C. Each primer was tagged with a unique 5' fluorescent dye, and PCR products from differently labeled primers were combined for fragment analysis on an ABI 3730 DNA analyzer. Fragments were analyzed using Genemapper^® ^software (Applied Biosystems, Foster City).

Four variable, consistently amplifiable, tetranucleotide microsatellite loci were developed (genbank accession #s: EU295522-EU295525, Table 1). A sample of 33 adult *G. flavescens *was used to characterize microsatellite loci. Each microsatellite locus was analyzed with GENEPOP version 3.4 [55] to calculate observed and expected heterozygosity and to test for Hardy-Weinberg equilibrium (Fisher's exact test). Genotypic disequilibrium for pairs of loci within the population (Fisher's exact test) was also assessed using GENEPOP.

### Parentage analysis

A Gentra PureGene™ cell and tissue kit was used to extract DNA from resident male caudal fin tissue. Genomic DNA was extracted from a random sample of larvae from each nest (48–96 larvae per nest) by placing individual larvae with sterilized forceps into separate wells of a 200 ul 96 well PCR plate containing a standard Proteinase K and 5% Chelex solution and digested for 1 hr at 55°C [56]. We used all four microsatellite markers to conduct parentage analysis on each nest. Embryos that consistently failed at one or more loci after two sequencing attempts were excluded from parental analysis. Embryos that failed to amplify all four loci were excluded from parentage analysis. Nest C08 consistently yielded poor amplification of microsatellite products, likely arising from sample DNA degradation, and was therefore excluded from parentage analysis. We calculated the number of offspring and undeveloped eggs per nest by averaging three replicate larval counts using volumetric sampling with replacement.

Exclusion probabilities and the minimum number of maternal genotypes that contributed to each nest were calculated using GERUD version 2.0 [57,58]. Female genotypes reconstructed with GERUD were matched using the Microsatellite Toolkit 3.1 for Microsoft Excel [59] and the probability of identity was estimated using LOCUSEATER [60]. All genotypes that were not consistent with the resident male's genotype (i.e. *de novo *mutations, miscalled alleles and sneaked copulations) were reamplified and analyzed for accuracy. If the genotype was still inconsistent with the resident male, the larva was excluded from GERUD analysis. We then found the minimum number of maternal genotypes that contributed to each nest using GERUD. Maximum likelihoods of maternal genotypes were determined using the "known parent" menu option in GERUD.

Genotypes excluded from parentage analysis were compared to the maximum likelihood maternal genotypes created by GERUD. If an excluded genotype deviated from the genotype of the resident male at only one locus, it was assigned as a *de novo *mutation/genotyping error. If a single larva failed to match at more than one locus, we assigned that larva to a second male. Homozygous genotypes of larvae were viewed with suspicion as these may represent null (non-amplifying) alleles in parental lines and were considered *de novo *mutations/genotyping error or sneaked fertilizations only if null alleles could be ruled out on the basis of the paternal and maternal genotypes. The number of embryos mothered by each female is proportional to the reconstructed maternal genotypes detected in a progeny array and is expressed as a proportion of the total embryos per nest.

### Statistical analysis

All data were analyzed first for normality and equal variances. Statistical tests are indicated throughout the text. All analyses were performed with JMP™ version 7.0.1 (SAS Institute Inc. Cary NC). Means are reported throughout the text ± the standard error of the mean (SE).

## Authors' contributions

KBM collected samples, developed the microsatellite primers, conducted all molecular analyses and drafted the manuscript. TA, EF and PAS collected samples and helped develop field techniques and animal husbandry aspects of the project. AGJ supervised molecular aspects of the study. All authors helped draft the manuscript and approved the final manuscript.

## Authors' information

KBM's research interests include fish behaviour, sexual selection and the evolution of genetic mating systems. TA and EF focus on sexual selection and parental care in fishes and birds, with a current emphasis on sex role dynamics and the evolution of female ornamentation. PAS is interested in fish behaviour, particularly reproductive behaviours. AGJ is interested in the use of molecular genetic techniques in the study of evolutionary processes.
